# Recommendations on palliative care aspects in intensive care medicine

**DOI:** 10.1186/s13054-023-04622-3

**Published:** 2023-09-18

**Authors:** Guido Michels, Manuela Schallenburger, Martin Neukirchen, ICU Palliative Study Group, Stefan John, Uwe Janssens, Philip Raake, Katharina Andrea Schütt, Johann Bauersachs, Thomas Barchfeld, Bernd Schucher, Sandra Delis, Rüdiger Karpf-Wissel, Matthias Kochanek, Simone von Bonin, Christiane M. Erley, Susanne D. Kuhlmann, Wolfgang Müllges, Georg Gahn, Hans Jürgen Heppner, Christoph H. R. Wiese, Stefan Kluge, Hans-Jörg Busch, Claudia Bausewein, Martin Pin

**Affiliations:** 1 Department of Emergency Medicine, Hospital of the Barmherzige Brüder, Trier, Germany; 2 Interdisciplinary Centre for Palliative Medicine, Heinrich-Heine-University Duesseldorf, University Hospital Duesseldorf, Düsseldorf, Germany; 3 Department of Anaesthesiology, Heinrich-Heine-University Duesseldof, Düsseldorf, Germany; 4 Center of integrated oncology Aachen, Bonn, Cologne (CIO ABCD) Heinrich-Heine-University, Düsseldorf, Germany

**Keywords:** Intensive care, Palliative care, Palliative medicine, Quality of life, Timely integration, Early integration

## Abstract

**Background:**

The timely integration of palliative care is important for patients suffering from various advanced diseases with limited prognosis. While a German S-3-guideline on palliative care exists for patients with incurable cancer, a recommendation for non-oncological patients and especially for integration of palliative care into intensive care medicine is missing to date.

**Method:**

Ten German medical societies worked on recommendations on palliative care aspects in intensive care in a consensus process from 2018 to 2023.

**Results:**

Based on the german consensus paper, the palliative care aspects of the respective medical disciplines concerning intensive care are addressed. The recommendations partly refer to general situations, but also to specific aspects or diseases, such as geriatric issues, heart or lung diseases, encephalopathies and delirium, terminal renal diseases, oncological diseases and palliative emergencies in intensive care medicine. Measures such as non-invasive ventilation for symptom control and compassionate weaning are also included.

**Conclusion:**

The timely integration of palliative care into intensive care medicine aims to improve quality of life and symptom control and also takes into acccount the often urgently needed support for patients’ highly stressed relatives.

## Background

Palliative care is an integral part of intensive care. While the german S3 guideline on palliative care for patients with incurable cancer, which has been extended in 2020, exists for incurable oncological patients [1, 2] there is no corresponding recommendation for non-oncological patients with life-limiting diseases. Although these patients are more often transferred to the intensive care unit (ICU) at the end of their lives [3]. However, recommendations for end-of-life care are important tools for comprehensive and qualitative care [4]. In addition to the intensive care therapy goals, palliative care focuses its efforts on maintaining or even improving the quality of life of patients and their relatives [5].

Palliative care can provide support not only in symptom reduction, patient and family communication, advance care planning (ACP), supportive care for relatives and, if necessary, palliative sedation in case of refractory symptoms [6, 7], but also implementation of psychosocial and spiritual care for patients and their relatives. All that may also prevent moral distress of staff [8, 9]. Health care workers can also experience a reduction in moral distress in difficult situations if they themselves are trained in palliative measures and carry them out [10].

In order to ensure palliative care 24 h/365 days a year, it is necessary, regardless of the availability of specialized palliative care, to ensure at least generalist palliative care on both the physician and nursing level in every clinic [6]. Employees in intensive care wards should receive a basic qualification as part of regular training. Basic knowledge of palliative care such as symptom relief, communication and end-of-life care should be taught and developed as an in-house standard operating procedure (SOP).

## Methodology and aims of the consensus paper

The purpose of this consensus paper is to implement evidence-based and consensus-based recommendations for a high-quality implementation of palliative care aspects in intensive care. The consensus paper was concentrated by ten german professional societies (German societies of Medical Intensive Care and Emergency Medicine, Cardiology, Hematology and Medical Oncology, Nephrology, Neuro-Intensive Care and Emergency Medicine, Anesthesiology and Intensive Care Medicine, Interdisciplinary Emergency and Acute Medicine, Palliative Medicine; Germany Respiratory Society; German Geriatric Society). The before-mentioned professional societies participated in the consensus process, which was initiated in 2018 and completed in 2023. The respective professional societies dealt with key issues in palliative medicine; finally, a specialist evaluation took place, in which recommendations were made.

The long version of the consensual recommendations with detailed explanations can be viewed in the german online publication (https://doi.org/10.1007/s00063-023-01016-9) [11].

## Results and recommendations

As part of the recommendations on palliative care aspects in intensive care, the focus was placed on eight subject-specific focal points [11].

### General recommendations

Based on the described advantages of palliative care, it can be said that palliative care should be integrated into intensive care at a timely stage. Thus, a specialized palliative care team should ideally be consulted for palliative counselling and subsequent palliative care treatment in potentially life-limiting situations for highly symptomatic intensive care patients and their relatives. In order to treat general symptoms, and especially, if no specialized palliative care service is available, intensive care physicians and nurses should have a basic palliative care qualification.

**Table:** 

Basic knowledge can be imparted regularly in the context of interdisciplinary and interprofessional training. In-house standard operation procedures (SOP) for common symptoms can also be developed and made available to staff.	

### Components of palliative care

In the palliative care team's co-treatment of intensive care patients with malignant and non-malignant primary diseases, the main tasks are to ensure good symptom control on four levels (physical, psycho-social and spiritual), ACP, as well as patient- and relatives-centered communication. The palliative care team can help to ensure this despite the highly technical environment of an ICU.

The content of discussions also lies in the determination of the patient's will. Due to the severity of the disease in the acute situation, intensive care patients are not able to express themselves. In such situations, it is good if advance directives are available. However, this is often not the case or they hardly refer to the acute situation. It is not always possible to discuss them with relatives who have power of attorney and to adapt them to the current situation. Therefore, a moderated discussion process in the sense of ACP should be recommended to patients and relatives before elective interventions and interventions where a subsequent intensive care treatment cannot be excluded.

For patients who have been under intensive care treatment for more than a week, therapy goal discussions should take place at least once a week with the relatives and, if possible, with the patients, even if there is a written will. These discussions should focus on the current condition, realistic therapy goals [12] and further care in the ICU. In case of ambiguity, information can be better communicated and new and adapted treatment goals can be set with the help of participatory decision-making [13, 14]. Patient will, values and goals should be taken into account as well as current medical evidence [15]. Questionable or doubtful medical indications should lead to increased discussions with those affected in order to jointly examine the meaningfulness [16].

Such discussions, but also time pressure, a high sense of responsibility as well as fear of failure and different attitudes in the team can make the treatment of dying patients in the ICU a great burden.

**Table:** 

An open culture of discussion in the multiprofessional team as well as multiprofessional decision-making, regular team meetings and supervision as a routine part of everyday work can ease the burden of intensive care teams.	

### Palliative care aspects in geriatric patients

The demographic trend as well as the increase in chronic diseases and the simultaneous progress in medicine mean that the proportion of older patients in hospitals at all healthcare levels will increase. Thus, they are also becoming an increasingly frequent part of acute care and it is difficult to determine the right time for a change in therapy goals. Therefore, a procedure should be discussed with the patients early in the process of treatment, which can and will be adjusted repeatedly. Discussions on changes in therapy goals should be held when the disease is in a compensated status, so that the patient has the opportunity to grasp the further progression of the disease and the resulting consequences.

The transitions from curation to palliation can often be rather fluid. A reliable assessment of the prognosis is not always possible due to various influencing factors. An important and influential factor here is frailty, which is associated with more difficult convalescence and higher mortality in geriatric patients [17]. The clinical frailty scale [18] is suitable as an additional assessment aid and should therefore also be used even in intensive care.

**Table:** 

The clinical frailty scale should be used for all intensive care patients. Advanced age alone is not a reason to refrain from treatment in the ICU.	

### Palliative care aspects in patients with terminal heart failure

Despite significant improvement in the therapy of heart failure, progression of the disease still occur. For an appropriate therapy, it should be checked which indications are present and the patient's will should be taken into consideration. So-called device systems are often used, and deactivation can be considered, especially at the end of life. Such a highly sensitive and individual decision can only be made together with the patient or his/her legal representatives. For this purpose, sufficient and individual information should be provided in advance and at an early stage.

**Table:** 

It is recommended that palliative medicine is routinely involved before the implantation of cardiac devices in terminal heart failure and that the individual procedures are determined within the context of ACP.	
Deactivating immediate life-sustaining technical support systems is always a very difficult and burdensome decision-making situation and should therefore be discussed in ACP before implantation.	

### Palliative care aspects in ventilation therapy

Invasive, as well as non-invasive ventilation (NIV) therapies, are essential components of intensive care. Both ventilation therapies are used in critical care. Most patients can be weaned off ventilation after the acute situation, but in about 20% this is continued. This is called prolonged weaning. Advanced age, especially frailty, and comorbidities can contribute to this. For some of the patients, weaning in special weaning centers is possible, but for others this also fails. In these patients, permanent non-clinical invasive ventilation becomes necessary. The usefulness of ventilation therapy should therefore be clarified at the time of initiating the therapy.

Ventilation therapies thus basically represent a bridging of an acute or acutely worsening disease situation. However, treatment goals and a quality of life corresponding to the patient's wishes cannot always be achieved. Therefore, a regular assessment of the medical indication should be carried out in the case of prolonged ventilation. In addition, after a certain duration of ventilation, the specialized palliative care team should always be consulted in the sense of timely integration. If the intended therapy goals cannot be achieved or if the treatment does not correspond to the patient's wishes, a change of therapy goals should be discussed with the patient. Termination of ventilation may also be possible here.

If the decision is made to end ventilation, relatives should be accompanied and well informed about possible physical reactions. Symptoms such as anxiety or dyspnea may occur and are the focus of symptom relief. Opioids can be used to reduce dyspnea sometimes in combination with benzodiazepines to reduce anxiety. If symptom control is not achieved, targeted sedation reduces symptoms of dyspnea and anxiety. Dose finding is done with regular monitoring of symptom burden.

**Table:** 

Ventilation therapy should be discontinued if the desired therapeutic goal cannot be realistically achieved or is not desired by the patient. Communication should be transparent, empathic and authentic, both in the team and with patients and relatives.	
Upon discontinuation of respiration, an adequate dose of opioid therapy for the prophylaxis of dyspnoea and benzodiazepine therapy for the prophylaxis of anxiety in the context of targeted sedation should be performed. Any shortening of life due to unavoidable side effects should be tolerated.	
Family members should be informed about possible physical reactions of the patient to the discontinuation of ventilation and accompanied accordingly.	
The responsible physicians should personally conduct and accompany the implementation of both the immediate extubation and the initiation of Compassionate Weaning. This task should not be left to the nursing staff alone. Physicians' very own task is to alleviate symptoms also in the dying phase.	

### Ventilation therapy by patients with advanced non-oncological diseases

In patients with advanced pulmonary, cardiac or neurological diseases, acute or acutely decompensating chronic respiratory insufficiencies may occur, which can generally be treated with ventilation therapy. The start of invasive ventilation therapy should be critically evaluated in these patients with regard to the possible medical prospects of success, the threat of intensive long-term therapy and the patient’s will. The use of temporary NIV can be a sensible option in terms of a curative approach [19].

NIV can also be continued or initiated in patients in whom intubation is not medically indicated because of advanced disease or who refuse intubation. Here, it should be ensured that NIV does not lead to more side effects and that a dying process is not unnecessarily prolonged.

**Table:** 

In patients with advanced pulmonary, cardiac or neurological diseases, NIV may be a treatment option when intubation with prolonged intensive care stay is not indicated or desired.	
The use of the NIV should not prolong an already initiated process of dying.	

### NIV for symptom reduction

The use of NIV including high-flow oxygen therapy can be used in an acute situation to relieve dyspnoea, but should be the last option [20]. The potential benefit must be critically weighed against possible adverse effects, such as respiratory dehydration or worsening of dyspnoea.

**Table:** 

NIV can be used as a palliative intervention to reduce symptoms of dyspnoea.	

### Patients with home mechanical ventilation

Home mechanical ventilation, which is usually necessary after unsuccessful weaning, is an increasing treatment option for patients with chronic respiratory insufficiency. Patients receiving invasive or NIV in the home setting often have to return to hospital in acute situations. It has been shown that quality of life and happiness is significantly reduced in such patients after one year of long-term survival [21]. The suffering of the affected patients can thus be unnecessarily prolonged and a dignified death prevented.

Patients should therefore receive routine and standardised outpatient palliative care.

If there is no hope of improvement or no longer any purpose for patients with home mechanical ventilation, a change of therapy goal with possible termination of ventilation therapy should be discussed and implemented with the patient and relatives [22].

**Table:** 

Patients receiving home mechanical ventilation therapy should receive outpatient palliative care as part of their home care.	
The indication for continued long-term ventilation should be assessed individually, critically and ideally independently, considering the patient’s prognosis and quality of life in the course.	

### Palliative care aspects in patients with terminal lung disease

Chronic obstructive pulmonary disease (COPD) is one of the leading diseases worldwide in terms of morbidity and mortality [23, 24]. Each acute exacerbation is associated with increased mortality, and a higher frequency of exacerbations further increases mortality. In addition to acute therapy, open communication with patients, if possible, and relatives is a precondition for comprehensive support. This may also include discussions about death or the preferred place of death. The termination of respiratory therapy under palliative care support should not be a taboo subject, but a natural offer [24].

Timely integration of palliative care, therefore, makes sense here as well. However, in COPD patients, due to the unpredictable course of the disease, it is difficult to determine the appropriate time for palliative care. Primary care teams, including those in the ICU, should therefore be trained in the treatment of critically ill COPD patients and fulfil the requirements of general palliative care [25].

Idiopathic pulmonary fibrosis is a severe and progressive disease of unknown cause. Affected patients have distressing symptoms such as dyspnoea or cough early in the progression of the disease. Early integration of palliative care therefore makes sense here [26–29].

Lung cancer remains the most common cause of death among all cancers. Expanded and improved diagnostics in recent years, as well as additional treatment options, have significantly improved survival [30]. As a result, patients with incurable lung cancer have to be cared for more frequently in ICUs. Therefore, possible treatment options and the need for acute intensive care should be discussed early in the course of the disease. If the patient is willing, it is possible to carry out intensive medical therapy for a certain period of time without restrictions (time-limited trial). In this case, both prognosis and therapy goals should be evaluated at the time of admission and again during the course of treatment. If there is no indication and the prognosis is bad, a therapy limitation with limited but symptom-reducing support can be offered after talking to patients and relatives.

**Table:** 

In patients with advanced underlying pulmonary disease and poor prognosis, palliative care should be provided with the aim of optimally alleviating symptoms by means of medication and non-medication measures.	
Discussion about the patient's death and preferred place of dying should generally be part of medical consultation. These issues should also be addressed in an acute situation as soon as the symptoms are reduced to the extent that the patient is able to do so.	
The termination of ventilation therapy under palliative care should be a matter of course in ICUs.	

### Palliative care aspects in patients with hypoxic ischemic encephalopathy and delirium

Hypoxic-ischemic encephalopathy (HIE) is the consequence of a reduced supply of oxygen to the brain, which is typically the result of a cardiac arrest. The assessment of HIE and thus the prognosis is multidimensional according to the currently valid guidelines. This includes cerebral imaging, electroencephalogram (EEG) and laboratory determination of neuron-specific endolase. If the prognosis assessment shows no prospect of recovery of cerebral functions and regaining consciousness, a therapy limitation should be discussed with the relatives. Comorbidities should also be considered.

If the EEG shows a treatable non-convulsive status epilepticus, but no other uncertain prognosis, an antiepileptic treatment should be attempted [31–33].

Epileptic seizures that occur during the course of the disease and that affect quality of life should be treated, even with a bad prognosis, but anticonvulsant therapy should not affect quality of life more than the seizures [34]. In palliative therapy, alternative modes of administration, such as buccal, intramuscular, subcutaneous or rectal, can be considered, even if it is an off-label use [35].

Both acute and palliative courses of illness offer multiple risk factors and causes for delirium. The state of delirium can be frightening and upsetting for patients, relatives and professional team members. Open communication and explanation of the clinical situation are therefore enormously important.

Even in the presence of primarily hypoactive delirium, an EEG can be performed to differentiate between a treatable non-convulsive status epilepticus. Before the use of drug therapies in delirious patients, possible general measures such as a calm environment that promotes orientation, prophylaxis against falls and calm communication should be carried out [2]. The indication for drug therapy should be based on the symptoms and the quality of life. Here, too, alternative forms of application can be used to a greater extent.

**Table:** 

Prognosis assessment after hypoxic ischemic encephalopathy (HIE) should be performed using a standard prognostic algorithm.	
If epileptic seizures are clinically or electroencephalographically detectable following an HIE, anticonvulsant therapy should be administered at a sufficiently high dose and for a sufficiently long period of time	
Patients with persistent consciousness disorder after HIE, as well as those with a suspected diagnosis of hypoactive delirium, should receive an EEG diagnosis in order not to overlook a potentially treatable non-convulsive status epilepticus.	
The therapy of delirium should basically consist of non-medicinal and medicinal components.	
Haloperidol can be an option for treatment of hyperactive delirium; there is currently no pharmaceutical therapeutic option for hypoactive delirium.	

### Palliative care aspects in patients with terminal renal diseases (dialysis requirement)

Up to one third of patients who require dialysis during their intensive care stay are dependent on renal substitution procedures at discharge [36]. Increasingly, chronic dialysis patients and patients with End Stage Renal Disease are also receiving acute medical treatment. These patients may be at increased risk of increased mortality, reduced quality of life and less likely to be discharged home [37].

As an alternative to machine renal replacement therapy, concepts for maximal conservative and palliative therapy have therefore been developed and investigated in recent years [38].

Due to the high physical symptom burden, palliative care is becoming increasingly important in ESRD patients. The palliative approach is patient- and relative-centered, with a focus on reducing symptom burden and suffering and improving quality of life and well-being. Thus, consensual avoidance of dialysis treatment and discontinuation of dialysis can become alternatives in this phase. Conservative management and symptom relief replace renal substitution procedures in the sense of a change of therapy goal.

Discontinuation should be discussed for patients who have a severely limited life expectancy, low quality of life, refractory pain or progressive deterioration due to an untreatable disease. This should take place in the sense of shared-decision-making in open and empathetic discussions.

At the end of dialysis, symptoms such as fatigue, sleep disturbances, dyspnoea, anxiety, pruritus and xerostomia, among others, should be controlled [39].

**Table:** 

In general, all patients in whom dialysis is stopped or deciding not to undergo dialysis should receive integrated palliative care.	
Patients who are initially offered a time-limited trial due to an uncertain prognosis should be offered integrated palliative care.	

### Palliative care aspects in patients with hematological/oncological diseases

Despite the many opportunities offered by new cancer therapies and the parallel positive developments in modern intensive care medicine, the limits of possible therapies must always be evaluated for cancer patients in intensive care. A good balance between what is possible, feasible and reasonable is urgently needed, so they should also be considered in the ICU from a palliative medicine perspective.

Due to the quite different prognosis, intensive medical treatment should, if possible, be discussed before admission to an ICU, in the best case already when the diagnosis is made. This must not be done against the will of the patient, his authorised caregiver or the written wish. On the other hand, an explicit wish for treatment in the ICU does not mean that this should necessarily take place. Here, the indication is decisive. For example, patients in poor overall condition who have no other treatment options and dying patients should not be admitted to an ICU.

For patients for whom not all the information for a decision is available or can be clarified, intensive care should first take place for a limited period of time. Subsequently, the situation can be re-evaluated in cooperation with other professional care providers, such as oncologists or palliative care specialists.

**Table:** 

In principle, before a patient with cancer is admitted to an ICU, it should be clarified whether intensive care is medically indicated and desired by the patient or legal representative.	
Acute medical care (without limitation of resources) should be offered to all seriously ill cancer patients if the intended therapeutic objective could be compatible with the overall prognosis of the underlying malignancy.	
The patient's will or the information given by an authorised representative must be complied with if medically meaningful. If there is a lack of meaning, this should be communicated openly and empathetically with the affected persons, pointing out the alternatives.	
In order to improve the quality of end-of-life care for critically ill cancer patients, e. g. the discontinuation of treatment and monitoring or maintaining of dignity and individual wishes, there should be close cooperation with specialist palliative care.	

### Management of palliative emergencies

In palliative emergencies, it is important to consider the different dimensions—medical, legal, spiritual, ethical, psychosocial—as these can influence the emergency as a whole [40]. Common symptoms leading to palliative emergencies can be respiratory distress, seizure disorder/ loss of consciousness, pain exacerbation, acute bleeding tendency, psycho-social crisis or cardiopulmonary resuscitation. The main focus is on a very good symptom-relieving therapy in order to maintain as high a quality of life as possible. The patient's problem as well as the cause should be reduced if possible. The prognosis and the patient's will should be included in decisions.

**Table:** 

Intensive care physicians should be familiar with the specifics of the “palliative emergency” and acquire basic knowledge in palliative care.	

## Conclusions

The consensus paper on palliative care aspects in intensive care medicine is intended to contribute to quality assurance in intensive care. Timely integration of palliative care can lead to shorter ICU and hospital stays, reduced treatment costs, improved symptom reduction (quality of life), increased satisfaction of patients, relatives and staff, and improved quality of overall medical care. However, mortality remains unchanged [41–46] or was reduced in the studies [47, 48].

## Data Availability

Not applicable.

## Abbreviations

ACP: Advance care planning
ICU: Intensive care unit
SOP: Standard operating procedure
COPD: Chronic obstructive pulmonary disease
EEG: Electroencephalogram
HIE: Hypoxic-ischemic encephalopathy
NIV: Non-invasive ventilation
